# Enhancing Thermal Insulation Property and Flexibility of Starch/Poly(butylene adipate terephthalate) (PBAT) Blend Foam by Improving Rheological Properties

**DOI:** 10.3390/polym17020138

**Published:** 2025-01-08

**Authors:** JunGi Hong, Junhyuk Lee, Sung Kyu Kim, Dasom Son, DongHo Kang, Jin Kie Shim

**Affiliations:** 1 Korea Packaging Center, Korea Institute of Industrial Technology, Bucheon 14449, Republic of Korea; 2 Department of Energy Engineering, Hanyang University, Seoul 04763, Republic of Korea; 3 Department of Chemical and Biological Engineering, Korea University, Seoul 02841, Republic of Korea

**Keywords:** starch-based foam, PBAT, rheology, thermal insulating, foaming behavior

## Abstract

Starch foam has attracted significant attention as an alternative to expanded styrene (EPS) foam owing to its abundance and biodegradability. Despite these merits, its limited thermal insulation and flexibility compared to EPS have hindered its utilization in packaging. Herein, we report the effect of blending with starch/PBAT on foaming behavior and physical properties during foaming processing. We fabricated a starch/PBAT blend with systematically controlled blending ratios (0, 10, 15, 20, and 25 wt%) to analyze their effect on the interaction and characteristics of blended foam. The blending of starch and PBAT significantly reduced complex viscosity, enhancing resin flow during the foaming process. This improvement in resin flow led to increases in expansion ratio while reducing density and cell wall thickness. The thermo-insulation performance improved to 0.043 W/mK with 20 wt% of PBAT due to the enhanced expansion ratio and cell morphology. Additionally, the flexural strain at break improved significantly from 2.8 ± 0.6% to 9.6 ± 1.0% with increasing PBAT content. Enhanced water resistance was also observed, demonstrated by a reduction in water absorption and an extension of dissolution time. Overall, these findings underscore the potential of starch/PBAT foam to improve the thermal-insulating property, flexibility, and water resistance while maintaining their biodegradability and sustainability.

## 1. Introduction

The development of sustainable and eco-friendly insulation materials has gained attention as a substitute for expanded polystyrene (EPS) foam due to its limitations such as its long decomposition duration during disposal, emission of toxic gasses during burning, and challenges in recycling [1,2,3,4,5,6]. Especially, starch foam has emerged as a sustainable alternative to petroleum-based EPS due to its eco-friendliness, cost-effectiveness, and abundant nature [7,8,9]. Recently, the incorporation of various plasticizers to starch foam has become an area of interest for overcoming the limitations of starch foam, such as low thermal insulation, poor processability, low mechanical properties, and high sensitivity to water [5,9,10,11,12]. Incorporation of plasticizers into starch can result in an increase in the thermal insulation property as well as an enhanced processability due to a decrease in viscosity during the foaming process [13]. Adding plasticizers enhances the thermal insulation of starch foams by increasing their ability to trap air within the matrix, which reduces thermal conductivity. For example, Da Róz et al. investigated the effect of various potential plasticizers added to starch via melt processing, demonstrating that the presence of hydroxyl functional groups of plasticizers improves the mechanical property of thermoplastic starch [14]. However, the potential for migration, which can lead to contamination of adjacent materials and for volatility, which can lead to a decrease in the mechanical performance over the long term has remained [15,16].

Blending various polymers is a widely used and effective strategy to produce high-performance materials that would otherwise exhibit inferior properties [17,18]. Combining starch with biodegradable polymers can result in a material with improved mechanical performance [19], preserved biodegradability [20], and maintained low cost [21]. Especially, the reduction in sensitivity to the water of starch can be achieved by blending hydrophilic starch with a hydrophobic biodegradable polymer [22,23]. The introduction of hydrophobic components such as PBAT into starch foam reduces its water sensitivity by creating a moisture-resistant barrier that limits water penetration and absorption, significantly enhancing the durability and utility of the starch-based materials in humid conditions. Also, the deficient mechanical properties of starch can be complemented by selecting an appropriate biodegradable polymer which has a mechanically superior property [9,24,25,26]. For instance, Ke et al. conducted a study that showed that blending starch with PLA enhances tensile strength, breaking elongation, and reduces water absorption [27]. Popescu et al. tried improving the mechanical properties, along with water resistance, of polyvinyl alcohol (PVA) [28]. However, to the best of our knowledge, achieving high thermo-insulation and flexible properties of starch-based foam through blending of the starch with biodegradable polymers has not yet been reported.

**Figure 1:**
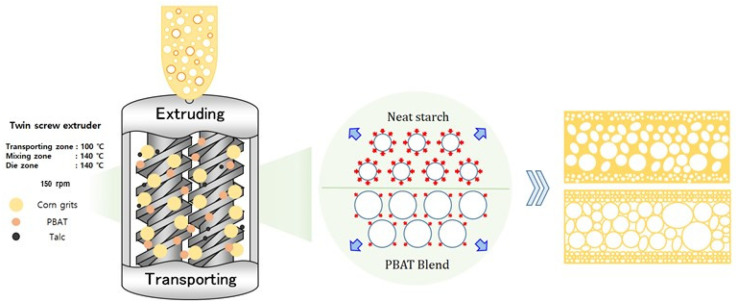
Schematic of extruding the starch blend foams and the comparison of neat starch and blend foams foaming process.

In this study, we demonstrated a model system of starch/bio-degradable blend foam to improve low thermal-insulation, brittle properties, and water-sensitive properties. We developed a starch/poly(butylene adipate terephthalate) (PBAT) blend foam, which has a low complex viscosity, flexible, and hydrophobic properties. The PBAT played two important roles in this system, namely (1) the viscosity reduction agent within the extruder and (2) a flexibility enhancer of composite foams (Figure 1). The correlation between mechanical properties of the foam and the rheological properties of the blends was investigated by fabricating starch/PBAT blend foams with various ratios of PBAT (0, 10, 15, 20, and 25 wt%). The blending of starch and PBAT decreased complex viscosity and improved resin flow during the foaming process. The enhanced resin flow made it easy to foam the starch/PBAT blend, resulting a high expansion ratio, low density, and improved thermo-insulation performance, while showing a minimum thermal conductivity 0.043 W/mK at PBAT 20 wt%. In addition, the starch/PBAT composite enhanced its flexural strain at break and compressive resilience owing to the flexible nature of PBAT. Furthermore, the introduction of PBAT enhanced water resistance, which is evidenced by the reduced water absorption and increased dissolution time in water.

## 2. Experimental Section

### 2.1. Materials

Corn grit was purchased from Dong-il FnG Co., Ltd. (Ansan-si, Republic of Korea). Poly(butylene adipate-co-terephthalate) (PBAT) (BG1000) was supplied by S-Enpol (Wonju-si, Republic of Korea). Talc supplied by Daejung Chemicals & Metals Co., Ltd. (Siheung-si, Republic of Korea) was used as the nucleating agent. Distilled water was used as both a plasticizer and a blowing agent.

### 2.2. Sample Preparation

The formulations and sample names of the mixture for foaming are listed in Table 1. First, we made the pre-mixture necessary for foaming by weighing and mixing all the ingredients in the bowl before putting them in the hopper and storing them for 30 min. We adjusted the PBAT content to 0 wt%, 10 wt%, 15 wt%, 20 wt%, 25 wt% and changed the moisture content to 13 wt%, 15 wt%, 17 wt%, 19 wt%, respectively. Then, pre-mixtures were extruded using a twin-screw micro-compounder (TSC 42/6, Brabender, Duisbur, Germany; length/diameter ratio = 6; screw diameter = 42 mm). The barrel temperatures with respect to the feeding zone were 100, 140, and 140 °C, and the screw rpm was set to 150. Then, the foam from the extruder was stabilized and samples were taken. The optimal feed water content according to the PBAT contents was determined by comparing the expansion ratio and cell wall thickness (Figures S1 and S2). Samples were named according to the composition that intuitively show the content of PBAT and feed water. The sample name P0W13 is a simple example, which presents a PBAT content of 10 wt% and feed water of 13 wt%.

### 2.3. Characterization

#### 2.3.1. Scanning Electron Microscopy (SEM)

SEM (SU8020, HITACHI, Tokyo, Japan) was used to observe the cross-section of corn grit foam cells. Foam specimens of about 3 mm thick were cut from a cross-section. The samples were coated with platinum using an ion sputter (E-1045, HITACHI, Tokyo, Japan) before the observation. The cross-section of each foam sample was measured under 30× magnification at 1 kV and 10 μA, and the scale bars indicated on the SEM images are 1 mm long. A lower voltage of 1 kV was used during the test in order not to damage the cell structures.

#### 2.3.2. Expansion Ratio, Density

The expansion ratio(ER) of the starch-based foams was determined as the square of the ratio of foam radius (*D*) to die radius (*d*). The result was the mean of five samples of each formulation. The density of foam was calculated as the relationship between weight and volume [29]. Reported values are averages of five determinations for each formulation.

(1) $$Expansion\;Ratio=\frac{D\left(mm\right)}{d\left(mm\right)}$$

(2) $$Density\left(g/{cm}^{3}\right)=\frac{w_{F}\left(g\right)}{V_{F}\left({cm}^{3}\right)}$$

#### 2.3.3. Rheological Properties

A rheometer (MCR 302, Anton Paar, Graz, Austria) was used to measure and compare the complex viscosity of each starch blend pre-foam at 140 °C at the approximate analysis conditions. In this case, all water content in the sample composition was substituted with glycerol because the water content of the samples affected the rheological characterization due to evaporation during analysis. The open nature of the rheometer plate caused the pre-foam made with water to foam during the test. It was assumed that the water molecules evaporated immediately and that only glycerol remained in the starch matrix during the heating (foaming) cycle [13]. Therefore, these substituted data could be reliable because the rheology of the foam matrix would be affected by the interaction of starch and glycerol according to our assumption. All measurements were conducted at a 1% strain, ensuring they were within the linear viscoelastic region (LVR) (Figure S3).

#### 2.3.4. Flexural Properties

Before mechanical testing, samples were conditioned for 24 h at a temperature of 25 °C and a relative humidity of 50%. Three-point flexural tests were carried out with the universal testing machine (Instron 3367, Instron, Norwood, MA, USA). The specimen diameter was 2.5 cm, span setting was 10.0 cm, and crosshead speed was 1 mm/min. The number of specimens of each sample was 5.

#### 2.3.5. Compressive Strength and Resilience

Before mechanical testing, samples were conditioned for 24 h at a temperature of 25 °C and a relative humidity of 50%. The mechanical properties of the foams were measured with the universal testing machine (Instron 3367, Instron, Norwood, MA, USA) equipped with a 30 kN load cell. The foams were fastened lengthwise and compressed by a probe of 6.0 mm diameter at a crosshead speed of 3 mm/min to a depth of 50% of the sample height [2,30]. The percent resilience was obtained by dividing the force required for a second compression by that for the first.

#### 2.3.6. Thermal Conductivity

The thermal conductivity of each sample was measured following ISO 22007-2 [31] using a hot disk (TPS-2500S, TA Instruments, New Castle, DE, USA) with an isotropic module, 2 s of measurement time, and 70 mW of heating power at 24 °C.

#### 2.3.7. Water Absorption and Water Resistance

The samples with 50.8 mm length and 15 mm thickness were used to determine the water absorption. Before the tests, the samples were dried at 80 °C for 24 h in a vacuum oven to determine the initial weight of each sample by removing the binding water in the samples. The dried samples were stored in a humidity chamber at 25 °C for 24 h under 70%RH, respectively. After storage in a humidity chamber, the samples were weighed to obtain the final weight with the water uptake. Then, the water absorption was calculated using Equation (3) as follows:

(3) $$Water\;absorption\left(\%\right)=\frac{w_{f}-w_{i}}{w_{i}}\times 100\%$$

where $w_{i}$ and $w_{f}$ are the initial weight before storage and the final weight after storage, respectively. The sustainability of the samples was characterized by dissolving the samples in distilled water. We dried the samples using same method for water absorption and dissolved 0.3 g of the samples in 200 mL of distilled water over time (after 1, 2, 3, 6, 12, 24, 48, and 64 h, and when completely dissolved) at 60 °C with magnetic stirring. We estimated the sustainability as the degree of dissolution of each sample using Equation (4).

(4) $$Degree\;of\;dissolution\left(\%\right)=\frac{w_{r}}{w_{d}}\times 100\%$$

where $w_{d}$ and $w_{r}$ are the weight of the dried sample and the weight of the remaining sample, respectively.

## 3. Results and Discussion

### 3.1. Preparation of TPS/PBAT Blend with Prepared Compatibilizers

Figure 1 illustrates a schematic diagram of the shape and the characteristics of the starch/PBAT blended foam. We designed the starch/PBAT blends in order to finely control the viscosity during the extrusion foaming process. The expansion of the starch/PBAT was conducted using a twin-screw micro-compounder (TSC 42/6, Brabender, Germany; length/diameter ratio = 6; screw diameter = 42 mm). The barrel temperatures with respect to the feeding zone were 100, 140, and 140 °C, and the screw rpm was set to 150. The foam samples were named according to the composition to intuitively show the content of PBAT (Table 1). During the foaming process, the water molecules acted as a plasticizer and penetrated into the starch molecule and gelatinized. After the gelatinization step, the water molecules evaporated rapidly under heat and pressure conditions, inducing cell expansion and the settlement of the final foam structures. In the case of neat starch foams, due to their instinct high viscosity, the foams cannot be smoothly expanded, producing a thick cell wall, high mechanical strength, and low thermo-insulation performance. On the other hand, in the case of the starch/PBAT foams, PBAT reduced viscosity within extruder, resulting in a thin cell wall and a large cell size.

### 3.2. Rheological Properties of Starch Blend Pre-Foam

**Figure 2:**
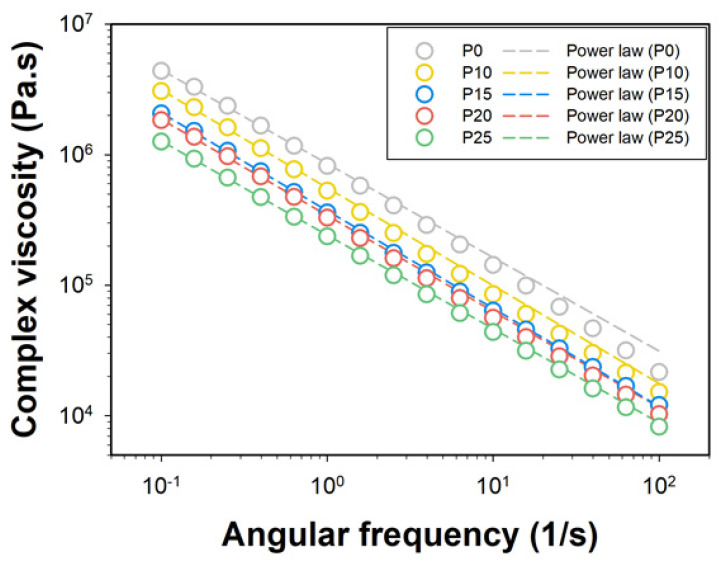
Complex viscosity of starch pre-foam with different PBAT contents at 140 °C.

To better understand the rheological behavior of starch/PBAT blends and their relevance to the foaming process, pre-foam samples were prepared using an internal mixer followed by molding with a hot press under conditions optimized for rheological analysis (Figure 2). These preparation methods ensured consistent sample quality for accurate rheological property measurements. The substitution of water with glycerol was employed to enable pre-foam preparation and rheological measurements, as water-based systems posed significant experimental limitations. It is important to note that the substitution of water with glycerol does not perfectly replicate the rheological behavior observed during actual extrusion foaming. However, this substitution allows for a representative analysis of how parameters vary under different PBAT blending ratios, providing valuable insights into the general trends and behavior of the blends. While absolute values of viscosity during extrusion foaming may differ, the observed trends in viscosity reduction, shear-thinning behavior, and resulting foam morphology remain relevant and insightful. The analysis revealed that the complex viscosity decreases as the content of PBAT increases. This decrease can be attributed to the role of PBAT as a viscosity thinning agent. This behavior aligns with findings from similar studies, such as those conducted by Huneault et al. (2011), who observed comparable trends in viscosity reduction in PLA/starch blends with the increasing concentrations of plasticizers [16]. The high viscosity of the matrix inhibits the cell expansion by moisture evaporation, reducing the size of the cell and thickening the cell wall, while the low viscosity matrix facilitates the cell expansion and thinning of the cell wall. To further understand the implications of these viscosity changes, Sandquist et al. (2019) demonstrated a clear correlation between viscosity measurements of non-foamed polymer blends and the size of foam cells developed during the foaming process, suggesting that rheological properties of the bulk material can predictably influence the cell morphology of the foam [32]. However, a viscosity drop that is too excessive caused the cell to fail to maintain its shape during temperature cooling process after cell expansion, resulting in a reduced cell size and a thick cell wall. Comparatively, Sun et al. (2011) also reported similar challenges in biopolymer blends, emphasizing the critical balance required in viscosity to achieve optimal foaming characteristics without compromising structural integrity [33]. The Power law model was applied to predict the flow curve of the blend with different contents of PBAT using Equation (5), and the model was compared with the experimental value [34].

(5) $$\eta^{*}={K\left|\omega \right|}^{n-1}$$

where $\eta^{*}$ is the complex viscosity and w is the angular frequency. The *K* is fitting parameters and *n* is *pseudo-plastic index*. Figure 2 presents both the experimental data (marked) and the power law model fit (dashed line), demonstrating a good fit between the model and the experimental values. The calculated power law parameters are shown in Table 2, where higher parameter values indicate stronger shear-thinning behavior. Among the samples, P0 exhibits the highest parameter values, likely due to the high molecular weight of starch.

Despite minor deviations at higher frequencies, Figure 2 illustrates that the power law model effectively captures the experimental data, confirming that the model accurately describes the viscosity behavior across the tested range.

### 3.3. Morphologies of Foam Cells in Each Starch Blend Foam

**Figure 3:**
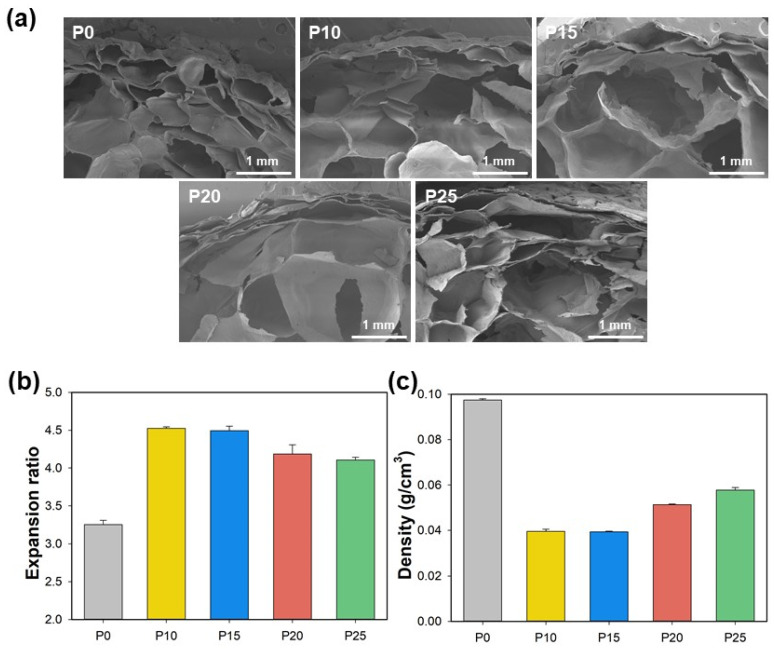
(**a**) Cross-sectional SEM images, (**b**) expansion ratio and (**c**) density of starch foam with various blending ratios.

Figure 3 shows the morphology, expansion ratio, and density of the starch/PBAT blend foam. In order to observe the interaction according to the PBAT content in the extrusion foaming process of starch, the cross-sectional SEM images of the starch blend foam were analyzed. The average cell size in the neat starch foam was 766 ± 259 μm, whereas in P10 and P15, the cells size increased to be 1497 ± 470 μm and 1703 ± 512 μm, respectively, and the cell wall became thinner. These results imply that PBAT lowered the viscosity of the starch/PBAT blend within the extruder during the foaming process. On the other hand, in the P20 and P25, it was confirmed that the cell wall became thicker again as the PBAT content increased. Even in the P25 sample, it was confirmed that the cell size also decreased to 1409 ± 424 μm. This means that due to an excessive decrease in viscosity, the cells lose theirs shape during the cooling process following cell expansion, leading to a decrease in cell size and a thickening of the cell wall [8]. This trend in the morphological behavior is consistent with the expansion ratio and density (Figure 3b,c), further supporting the notion that blend P15 exhibits optimal blend effects.

### 3.4. Mechanical Properties of Starch Blend Foam

**Figure 4:**
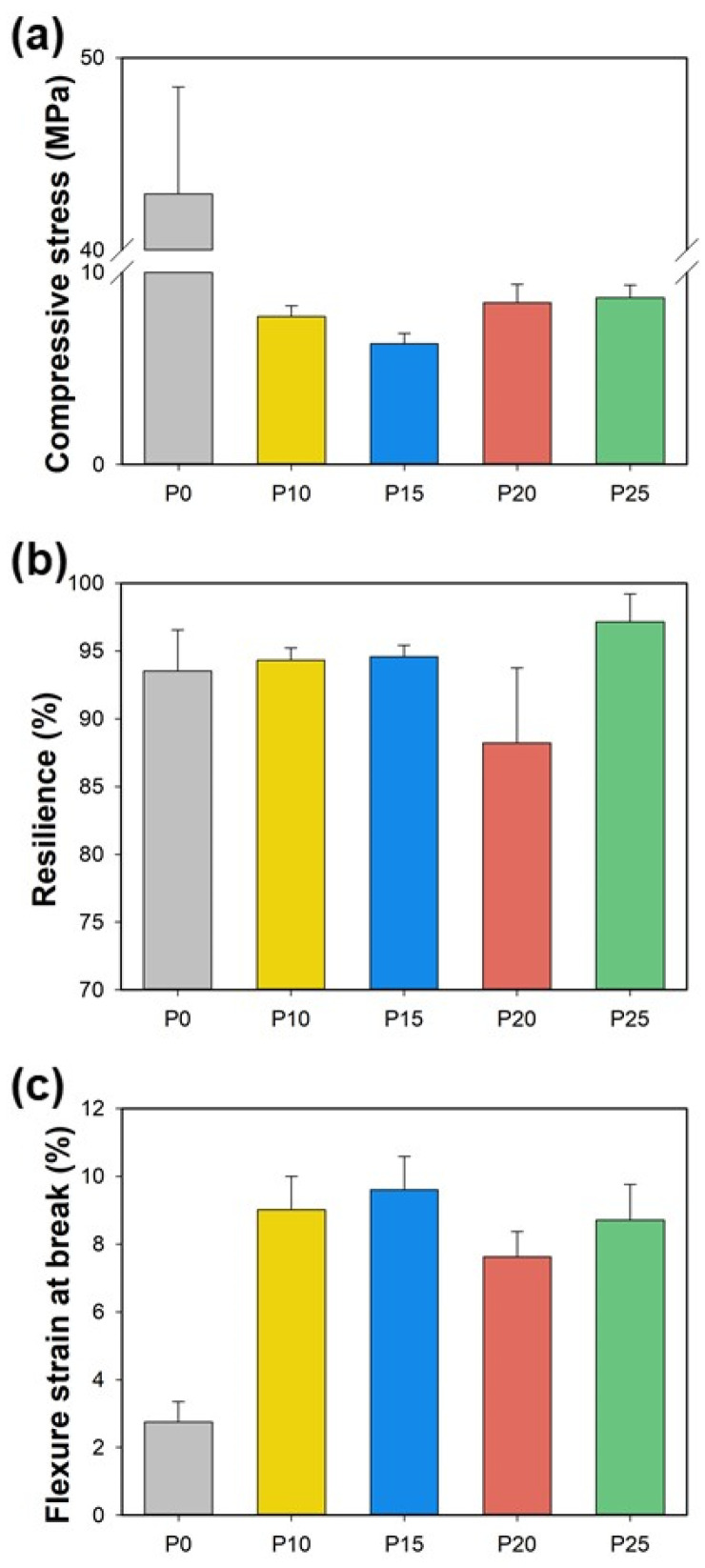
(**a**) Compressive stress, (**b**) resilience, and (**c**) flexural strain at the break of starch blend foams conditioned at 25 °C, RH 50%.

The influence of the starch/PBAT blend on mechanical properties was assessed through compressive and flexural tests, as depicted in Figure 4. Compressive strength was highest in the neat starch foam (P0), measured at 45.50 ± 3.10 MPa. A significant decrease in compressive strength was observed with the introduction of PBAT, indicating that mechanical resistance diminishes as the blend becomes less viscous and the cell walls thin. Specifically, in samples P10 and P15, where the PBAT content resulted in larger cell sizes and thinner walls, compressive strengths of 7.82 ± 0.22 MPa and 6.26 ± 0.34 MPa were recorded, respectively. However, in samples P20 and P25, which contained higher proportions of PBAT, there was a slight increase in compressive strength, reaching 8.53 ± 0.86 MPa and 8.32 ± 0.61 MPa, respectively. Flexural tests revealed that while higher compressive strength correlates with brittleness, the introduction of PBAT enhances flexibility. The flexural strain at break for neat starch (P0) was 2.8 ± 0.6%. This metric improved significantly in blends with PBAT, peaking at 9.6 ± 1.0% in P15, illustrating enhanced resistance to external loads. The resilience of the foam decreases up to sample P15, after which it increases again. This behavior can be attributed to the thinning of the cell walls, making them more susceptible to stress and prone to breaking easily. This observation is consistent with the morphological trend observed from samples P0 to P15, which exhibited a progressive thinning of the cell walls and an increased expansion ratio, and the lowest thermal conductivity up to sample P15. This analysis demonstrates a trade-off between compressive strength and flexibility, with optimal mechanical properties observed at intermediate levels of PBAT addition. These findings suggest that starch/PBAT blends offer a promising avenue for tailoring the mechanical performance of biodegradable foams to meet specific application requirements.

### 3.5. Thermal Conductivities of Starch Blend Foam

**Figure 5:**
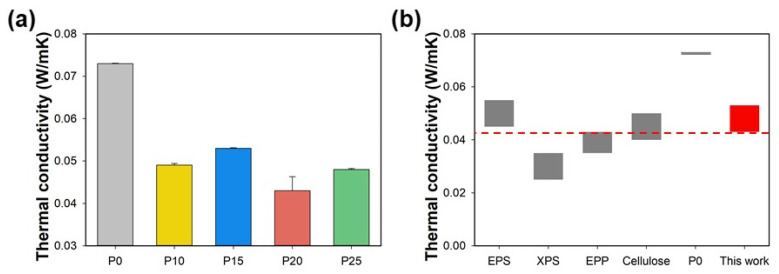
(**a**) Thermal conductivities of starch blend foams of various blending ratios, (**b**) comparison of thermal conductivities with commercial and other reported thermo-insulators with starch blend foams in this work.

The thermal conductivity of the starch/PBAT blend foams, as illustrated in Figure 5a, varied according to PBAT concentration. Neat starch foam (P0) exhibited a thermal conductivity of 0.073 W/mK, whereas the introduction of PBAT significantly lowered these values: P10 recorded 0.049 W/mK, P15 had 0.053 W/mK, P20 showed 0.043 W/mK, and P25 reported 0.048 W/mK. These changes in thermal conductivity do not precisely correlate with trends in morphology and expansion ratio, suggesting the uniformity and distribution of cells within the matrix. Non-uniform cell distribution can lead to variable path lengths for heat transfer, complicating the relationship between morphology and thermal properties. Figure 5b contextualizes the thermal performance of our starch/PBAT blend foams relative to established insulation materials like EPS, XPS, and EPP. The starch/PBAT foams demonstrate competitive thermal conductivity values, highlighting their potential as sustainable alternatives in thermal insulation applications. The effectiveness of the starch/PBAT blend in reducing thermal conductivity showcases the benefits of biodegradable composites in eco-friendly building materials, suggesting that optimal blending can achieve desirable thermal properties without compromising environmental sustainability. The increased flexibility and improved thermal insulation capabilities of starch/PBAT blends make them suitable for applications requiring durable yet lightweight materials, such as protective packaging, automotive parts, and biodegradable containers. This aligns with research by Gunathilake et al. (2022), who noted similar applications in their study of biodegradable foams where enhanced mechanical properties led to broader utilization in industrial sectors [3].

### 3.6. Water Resistance of Starch Blend Foam

**Figure 6:**
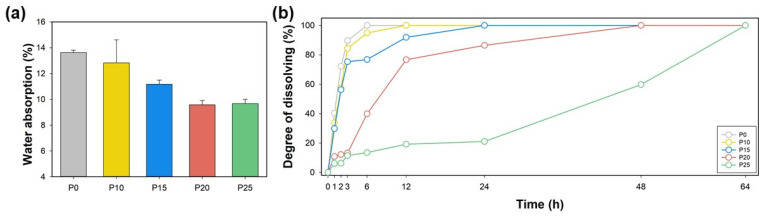
(**a**) Water absorption at 70%RH and (**b**) degree of dissolving at 60 °C water with different PBAT contents.

The water resistance of starch/PBAT blend foams was investigated by assessing water absorption and dissolution times, as represented in Figure 6. Neat starch foam demonstrated the highest water absorption at 13.63%, which progressively decreased with the incorporation of hydrophobic PBAT: 12.83% for P10, 11.18% for P15, and 9.58% for P20, and 9.67% for P25. Correspondingly, dissolution times in water at 60 °C extended significantly from 6.5 h for neat starch to 12.5 h for P10, 24 h for P15, 48 h for P20, and 64 h for P25. This reduction in water absorption and the extension of dissolution times can be attributed to the hydrophobic nature of PBAT. As PBAT content increases, the hydrophobic interactions within the foam structure become more dominant, leading to less affinity for water. To provide a clearer understanding of how PBAT incorporation impacts water resistance quantitatively, the rate of water absorption decreased by approximately 7% from P0 to P25, indicating a substantial improvement in hydrophobic properties due to the PBAT addition. Regarding the blending of starch and PBAT, the two components do not form perfectly homogeneous blends but rather exhibit phase-separated structures where PBAT domains are dispersed within the starch matrix. This phase behavior is crucial, as the dispersed PBAT domains interrupt the continuity of the hydrophilic starch matrix, effectively creating barriers that reduce water penetration and enhance the overall water resistance of the foam. This hydrophobicity not only decreases the amount of water the foam can absorb under relative humidity conditions (RH 70%) but also enhances its structural integrity in aqueous environments, delaying water-induced disintegration. Figure 6a shows the quantitative data on water absorption, indicating a clear trend of decreased absorption with increased PBAT content. Figure 6b details the prolongation of dissolution times, reflecting the enhanced water resistance of the blends. The enhanced water resistance of these starch/PBAT foams is particularly valuable in applications such as outdoor furniture, horticultural pots, and protective packaging for electronics, where resistance to moisture is crucial for the durability and longevity of the products. These applications benefit from the use of materials that can withstand environmental exposure without rapid degradation, thus reducing waste and the need for frequent replacements. In addition, comparing the environmental impact of these starch/PBAT blends to conventional packaging materials highlights their sustainability. While starch is a renewable resource that biodegrades, PBAT enhances the blend’s functional performance without compromising biodegradability. This combination offers a substantial reduction in environmental footprint compared to traditional petroleum-based foams like EPS, which are neither biodegradable nor sustainable. The potential for reducing pollution and waste through the adoption of these biodegradable foams is significant, making them an attractive alternative for industries looking to improve their sustainability practices.

## 4. Conclusions

In conclusion, this study successfully demonstrated the effects of blending starch with poly(butylene adipate terephthalate) (PBAT) on the properties of biodegradable foams intended for enhanced thermal insulation and flexibility. Systematic control of PBAT content within starch foams significantly influenced their physical, mechanical, and water-resistant properties. The addition of PBAT led to a notable decrease in complex viscosity, which facilitated better foam expansion, resulting in foams with lower density, reduced cell wall thickness, and improved thermal insulation properties. Notably, thermal conductivity decreased as the PBAT content increased, achieving as low as 0.043 W/mK with a 20 wt% PBAT blend. Mechanically, the inclusion of PBAT enhanced the flexibility of the foams, with flexural strain at break improving markedly across the tested ranges. Water resistance was also significantly improved, as evidenced by decreased water absorption rates and extended dissolution times in water, making these foams more suitable for applications requiring moisture resistance. Future research should focus on optimizing the formulation and processing conditions to further enhance the scalability and performance of these materials for industrial applications. Investigating the lifecycle impacts and the economic viability of large-scale production can facilitate the adoption of starch/PBAT blends in mainstream applications. Additionally, exploring the integration of natural fiber reinforcements might improve the mechanical properties and broaden the utility of these biodegradable foams in more demanding applications. These findings illustrate the potential of starch/PBAT blends not only as sustainable alternatives to conventional foams like EPS but also for enhancing the performance of starch-based materials to meet specific application requirements. The insights from this study provide a foundation for further exploration of starch-based composite materials, emphasizing the balance between biodegradability and performance in environmentally friendly packaging solutions. This research underscores the importance of tailored polymer blends in advancing the field of sustainable materials science, opening new avenues for the application of biodegradable foams in industries such as packaging, insulation, and beyond.

## Figures and Tables

**Table 1:** Sample code and contents of each corn grits foam.

Sample Name	PBAT (g)	Water (g)	Corn Grits (g)	Talc (phr)
P0	0	208.8	890	1
P10	93.7	201.5	890	1
P15	140.5	181.9	890	1
P20	187.4	161	890	1
P25	234.2	168	890	1

**Table 2:** Power law parameters of each corn grit foam.

Sample Name	Power Law Parameter (n)
P0	0.282
P10	0.250
P15	0.250
P20	0.264
P25	0.280

## Data Availability

Dataset available on request from the authors.

## Supplementary Materials

The following supporting information can be downloaded at: https://www.mdpi.com/article/10.3390/polym17020138/s1, Figure S1: Optimal feed water content according to PBAT content, Figure S2: Cell wall thickness of each starch blend foam samples, Figure S3: Dynamic strain sweep test results for starch (P0) and starch/PBAT blend samples. The left *y*-axis represents the storage modulus (G′) and loss modulus (G″), while the right *y*-axis represents the shear stress as a function of strain amplitude.
