# Supplementary material for: Enhancing Thermal Insulation Property and Flexibility of Starch/Poly(butylene adipate terephthalate) (PBAT) Blend Foam by Improving Rheological Properties
Source: Polymers (Basel). 2025 Jan 8;17(2):138. doi: 10.3390/polym17020138 (PMC11768926; doi:10.3390/polym17020138)
Supplement: Supplementary file 1 [file polymers-17-00138-s001.zip › polymers-3288395-supplementary.pdf]

Supporting Information

1. Optimal feed water content according to PBAT content

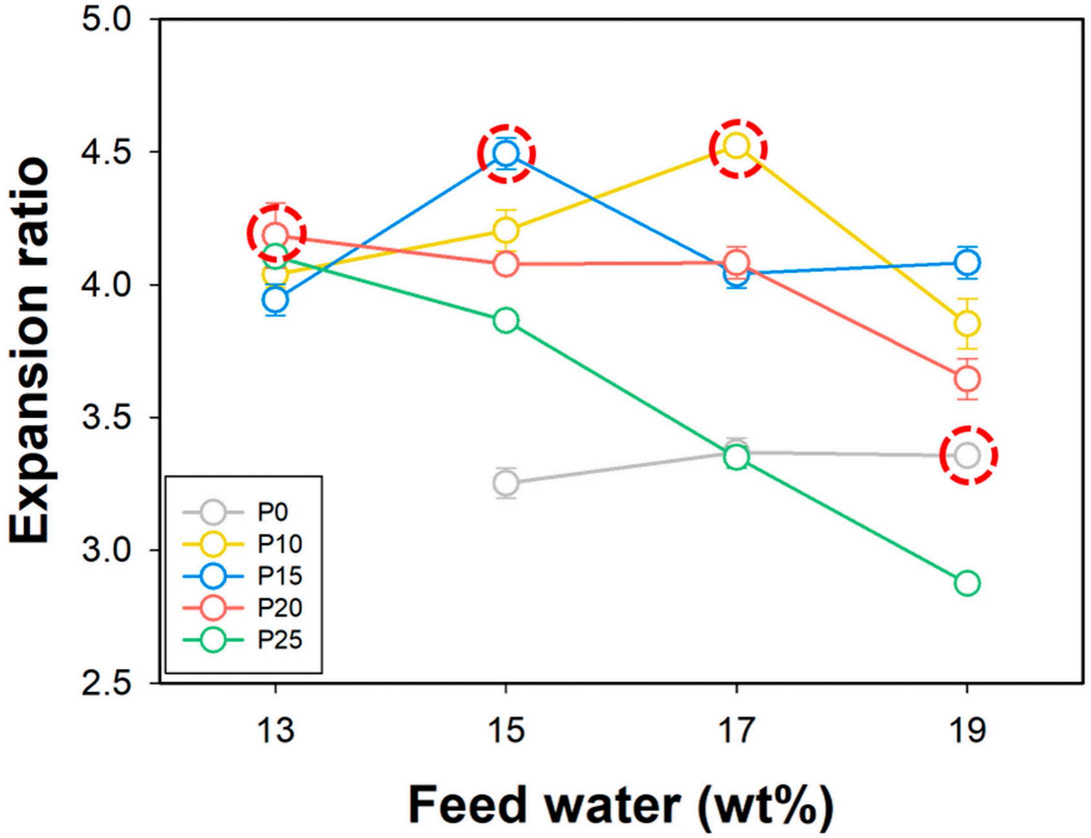

Figure S1. Optimal feed water content according to PBAT content

2. Cell wall thickness of each starch blend foam samples

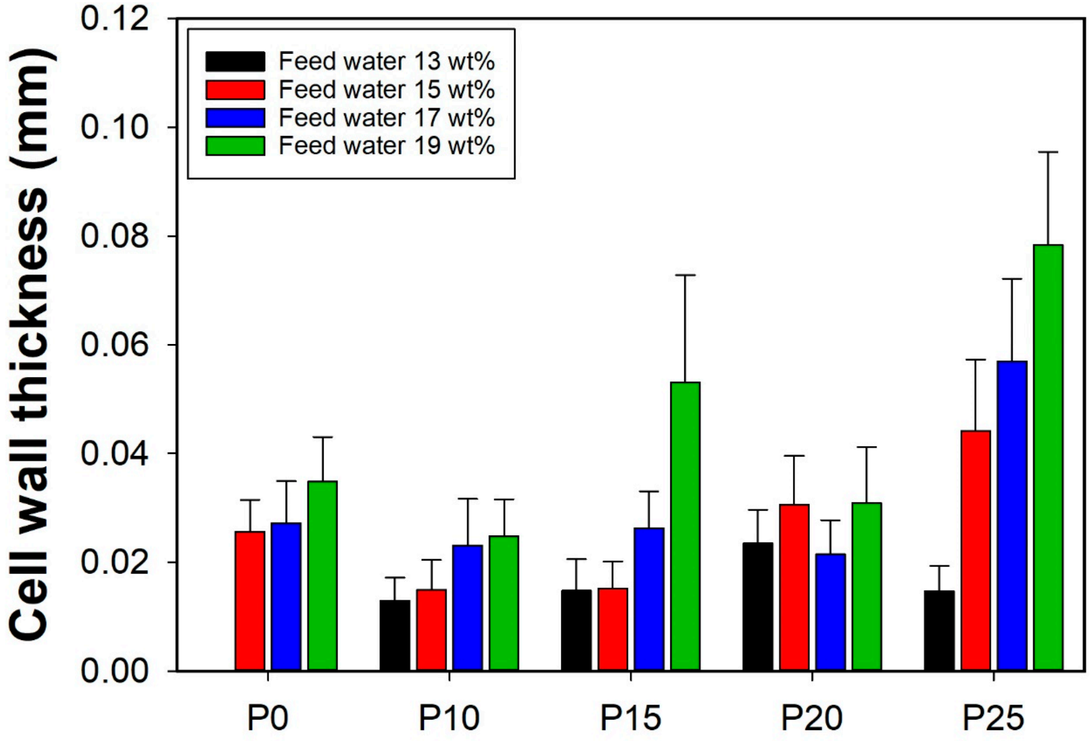

Figure S2. Cell wall thickness of each starch blend foam samples

3. Dynamic strain sweep test

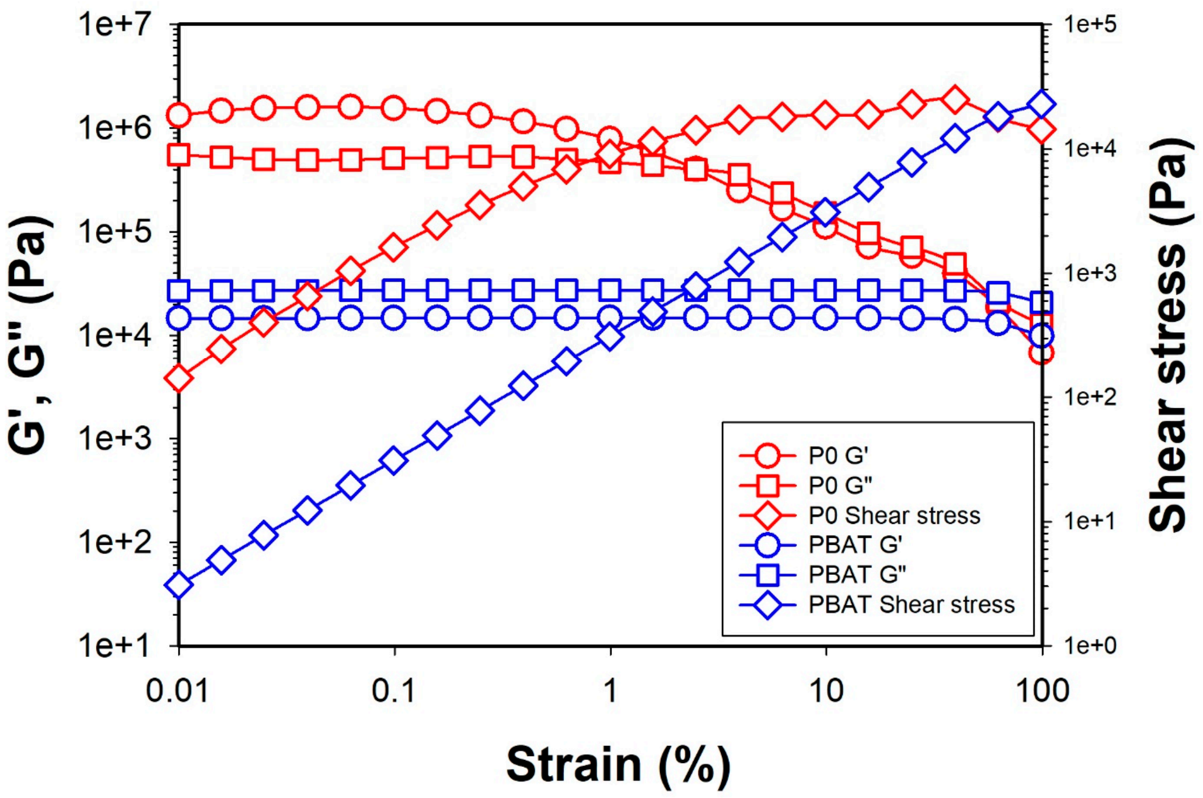

Figure S3. Dynamic strain sweep test results for starch (P0) and starch/PBAT blend samples. The left y-axis represents the storage modulus (G') and loss modulus (G"), while the right y-axis represents the shear stress as a function of strain amplitude.
